# Supplementary material for: Resolving the full spectrum of human genome variation using Linked-Reads
Source: Genome Res. 2019 Apr;29(4):635–45. doi: 10.1101/gr.234443.118 (PMC6442396; doi:10.1101/gr.234443.118)
Supplement: Supplemental Material [file supp_gr.234443.118_Supplemental_longranger-2.2.zip › longranger-2.2.tar.bz2!/lr2.2-public/lib/stan/cmdstan/stan/doc/api/html/arg__sample_8hpp_source.html]

Stan: src/stan/gm/arguments/arg\_sample.hpp Source File


|  |  |
| --- | --- |
|  | Stan  2.5.0  probability, sampling & optimization |


- Main Page
- Related Pages
- Namespaces
- Classes
- Files

- File List
- File Members

All Classes Namespaces Files Functions Variables Typedefs Enumerations Enumerator Friends Macros Pages

- src
- stan
- gm
- arguments

arg\_sample.hpp

Go to the documentation of this file.

1 #ifndef STAN\_\_GM\_\_ARGUMENTS\_\_SAMPLE\_\_HPP

2 #define STAN\_\_GM\_\_ARGUMENTS\_\_SAMPLE\_\_HPP

3

4 #include <stan/gm/arguments/categorical\_argument.hpp>

5

6 #include <stan/gm/arguments/arg\_num\_samples.hpp>

7 #include <stan/gm/arguments/arg\_num\_warmup.hpp>

8 #include <stan/gm/arguments/arg\_save\_warmup.hpp>

9 #include <stan/gm/arguments/arg\_thin.hpp>

10 #include <stan/gm/arguments/arg\_adapt.hpp>

11 #include <stan/gm/arguments/arg\_sample\_algo.hpp>

12

13 namespace stan {

14

15  namespace gm {

16

17  class arg\_sample: public categorical\_argument {

18

19  public:

20

21  arg\_sample() {

22

23  \_name = "sample";

24  \_description = "Bayesian inference with Markov Chain Monte Carlo";

25

26  \_subarguments.push\_back(new arg\_num\_samples());

27  \_subarguments.push\_back(new arg\_num\_warmup());

28  \_subarguments.push\_back(new arg\_save\_warmup());

29  \_subarguments.push\_back(new arg\_thin());

30  \_subarguments.push\_back(new arg\_adapt());

31  \_subarguments.push\_back(new arg\_sample\_algo());

32

33  }

34

35  };

36

37  } // gm

38

39 } // stan

40

41 #endif

42

stan::gm::arg\_sample

**Definition:** arg\_sample.hpp:17

stan::gm::arg\_sample::arg\_sample

arg\_sample()

**Definition:** arg\_sample.hpp:21

arg\_adapt.hpp

categorical\_argument.hpp

stan::gm::categorical\_argument

**Definition:** categorical\_argument.hpp:11

stan::gm::categorical\_argument::\_subarguments

std::vector< argument \* > \_subarguments

**Definition:** categorical\_argument.hpp:153

arg\_thin.hpp

stan::gm::arg\_save\_warmup

**Definition:** arg\_save\_warmup.hpp:10

arg\_num\_samples.hpp

arg\_num\_warmup.hpp

stan::gm::arg\_thin

**Definition:** arg\_thin.hpp:10

arg\_save\_warmup.hpp

stan::gm::argument::\_name

std::string \_name

**Definition:** argument.hpp:76

stan::gm::arg\_num\_warmup

**Definition:** arg\_num\_warmup.hpp:10

arg\_sample\_algo.hpp

stan::gm::arg\_num\_samples

**Definition:** arg\_num\_samples.hpp:10

stan::gm::arg\_sample\_algo

**Definition:** arg\_sample\_algo.hpp:13

stan::gm::arg\_adapt

**Definition:** arg\_adapt.hpp:19

stan::gm::argument::\_description

std::string \_description

**Definition:** argument.hpp:77


---

[ Stan Home Page ]

*© 2011–2014,
Stan Development Team.*
